# Comparison of Wound Healing Effects of Different Micro-Patterned Hydrogels on the Skin of Secondary Intention Rat Model

**DOI:** 10.3390/gels11040239

**Published:** 2025-03-24

**Authors:** Hong Jin Choi, Zeeshan Ahmad Khan, AbuZar Ansari, Jeonghyun Choi, Eun Jin Kim, Seo-Hee Han, Ho-Jun Song, Ok Chan Jeong, Yonggeun Hong

**Affiliations:** 1 Department of Digital Anti-Aging Healthcare, Graduate School of Inje University, Gimhae 50834, Republic of Korea; hjchoi@inje.ac.kr (H.J.C.); mickey970415@gmail.com (E.J.K.); sau12362@naver.com (S.-H.H.); songhojun02@gmail.com (H.-J.S.); 2 Department of Physical Therapy, College of Biomedical Science & Health, Inje University, Gimhae 50834, Republic of Korea; acezeeshan@live.com (Z.A.K.); jeonghyunch@dhu.ac.kr (J.C.); 3 Research Center for Aged-Life Redesign (RCAR), Inje University, Gimhae 50834, Republic of Korea; 4 Biohealth Products Research Center (BPRC), Inje University, Gimhae 50834, Republic of Korea; 5 Department of Rehabilitation Medicine of Korean Medicine, Dongguk University, Goyang 10326, Republic of Korea; abu.kim.0313@gmail.com; 6 Department of Biomedical Engineering, College of Biomedical Science & Health, Gimhae 50834, Republic of Korea; 7 Department of Rehabilitation Science, Graduate School of Inje University, Gimhae 50834, Republic of Korea

**Keywords:** hydrogel patch, micro-pattern, skin, wound healing, inflammation

## Abstract

Background: The skin acts as a barrier against external threats, and moisture is crucial for effective wound healing, as it promotes epithelial cell migration. Thus, a high water content supports wound healing by maintaining moisture, absorbing exudate, and forming a protective barrier. Here, we created three different micro-patterned hydrogels and tested them on rat skin wounds. Materials and Methods: Three different micro-patterned (waves, lines, and checks) hydrogel patches were created using three-dimensional polymer networks. On SD rat skin, wounds were created by making incisions, and the hydrogel patches were applied. The rats were divided into three experimental groups based on the hydrogel micro-patterns. Rats without hydrogel (vehicle) and those with flat hydrogel (no shape) were considered as controls. The wound closure rate (WCR) was calculated, and the expression of Col1A protein was measured by western blot. Results: After 7 days, the WCR was significantly higher in the groups treated with micro-patterned hydrogel patches compared to the vehicle and no-shape groups. Specifically, the WCR was highest in the checks micro-patterned hydrogel group compared to the waves and lines micro-patterned hydrogel groups. Furthermore, Col1A protein expression was evaluated at days 7 and 14, revealing a significant increase in expression after 14 days in the checks micro-patterned hydrogel group compared to the waves and lines micro-patterned hydrogel groups. Conclusions: The checks micro-patterned hydrogel patches demonstrated superior wound healing efficacy, as indicated by a higher WCR and increased Col1A protein expression after 14 days. These findings highlight the importance of hydrogel pattern design in improving wound healing suggesting that optimized micro-patterns can enhance therapeutic outcomes in skin wound management.

## 1. Introduction

The skin constitutes the outer barrier of the human body, providing comprehensive biological, chemical, and physical protection while also serving as a communicative organ that directly interacts with the external environment. As the largest organ of the human body, the skin performs multiple critical functions, including thermoregulation, sensory perception, and immune defense. This multifaceted role underscores the importance of maintaining skin integrity and health [1]. Given its crucial role, the skin is inherently vulnerable to various forms of damage, such as mechanical injuries, burns, surgical wounds, and chronic ulcers. These injuries can disrupt the skin’s protective barrier, leading to an increased risk of infection, fluid loss, and impaired functionality [2]. Consequently, effective skin wound management is paramount due to its impact on healthcare costs, healing efficacy, and increasing social demand.

The skin wound healing process is highly complex, involving overlapping stages of hemostasis, inflammation, proliferation, and remodeling [3]. This process is facilitated by the interaction of various cell types, such as keratinocytes, adipocytes, and fibroblasts, each contributing to different aspects of tissue repair and regeneration [4,5]. The effectiveness of wound dressings is pivotal in determining the speed and quality of recovery, making them an essential component of wound care [6]. In a dry environment, cells may lose functionality or die, thereby slowing the overall skin wound healing process. Thus, an ideal skin wound dressing should provide several key functions: protecting from external contaminants, maintaining a moist environment, promoting cell migration, allowing ease of application and removal, and minimizing interference with the healing process [7]. Maintaining a moist environment is particularly critical, as it prevents dehydration of the wound bed, reduces pain, and facilitates more efficient migration and proliferation of epithelial cells [8].

Hydrogels have emerged as a promising class of wound dressings due to their unique properties. Hydrogels are hydrophilic gels characterized by a three-dimensional polymer network that can absorb large quantities of water, thereby forming a moist barrier with the external environment [9,10]. This high water content supports autolytic debridement and provides a cooling effect, which can help reduce pain and inflammation in the affected area [11]. The structure of hydrogels, determined by the type of polymer used and the crosslinking method, offers various functional benefits, including antibacterial properties, self-healing capabilities, and responsiveness to environmental stimuli [11,12]. Commonly used hydrogel polymers include polyvinyl alcohol (PVA), polyethylene glycol (PEG), and chitosan (CS), all of which are known for their biodegradability and application in cosmetics, pharmaceuticals, and tissue engineering [13,14,15,16]. Despite their favorable properties, these materials often suffer from weak mechanical properties, necessitating frequent dressing changes and highlighting the need for improved physical bonding methods [17].

The micro-patterned surfaces of hydrogels, characterized by their shape, geometric dimensions, and relief height, play a crucial role in influencing cellular behaviors such as adhesion, migration, and proliferation. Studies have demonstrated that specific geometric patterns, such as circular or rectangular designs, along with variations in relief height, can modulate cell responses and enhance the wound healing processes [18]. The ability to precisely control these surface features during the stamping process ensures consistency and reproducibility in hydrogel fabrication. By tailoring these structural parameters, hydrogels can be engineered to create an optimal microenvironment that promotes key cellular activities crucial for tissue regeneration and repair. This strategic design approach ultimately accelerates wound healing and improves therapeutic outcomes.

Hydrogels can be classified in multiple ways based on their composition, structure, responsiveness to stimuli, application, and water content. This classification allows for the tailoring of hydrogel properties for specific uses, ranging from biomedical applications to agriculture and food technology. For instance, natural, synthetic, and hybrid hydrogels each offer unique advantages in terms of biocompatibility, mechanical strength, and functional versatility. Additionally, hydrogels can be designed to respond to different stimuli, such as temperature, pH, ions, and light, further enhancing their utility in dynamic environments [19].

This study focuses on the fabrication of hydrogels with different surface characteristics, evaluating their physical and biological properties to assess their potential as advanced wound dressings. By exploring a range of micro-patterned hydrogel surfaces, we aim to identify optimal designs that enhance the wound healing process. For this purpose, DOW CORNING 7-9700 Soft Skin Adhesive was selected due to its excellent biocompatibility, strong adhesive properties, and potential clinical applicability. To isolate the influence of surface properties on wound healing outcomes, this study deliberately emphasizes surface geometry rather than molecular structure or chemical composition. Ultimately, our findings contribute to the development of multifunctional, mechanically robust, and highly effective hydrogel-based wound dressings, addressing current limitations in wound care and meeting a critical clinical need to improve patient outcomes.

## 2. Results and Discussion

### 2.1. Hydrogel Patches Recover Wound Incision Rate

**Figure 1:**
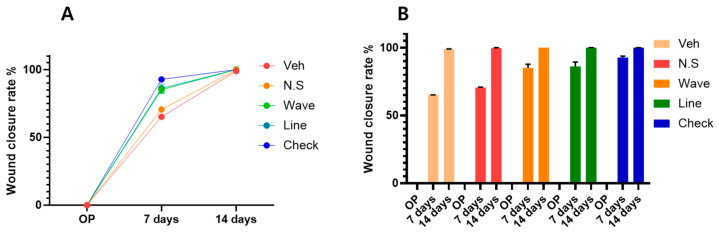
Wound closure rate of various hydrogel groups (Vehicle, no shape, wave, line, check micro-patterned group) at 7 days and 14 days. (**A**) profiles (**B**) bar diagram.

This study analyzes the effect of micro-patterned hydrogel on wound recovery by measuring the wound closure rate (WCR). The WCR reached 65.1% in the vehicle group, 70.5% in the no-shape group, 78.2% in the waves group, 76.8% in lines, and 90.4% in the checks micro-patterned group at day 7 (Figure 1A). Notably, after 14 days, the WCR was similar in all groups, reaching 98.8% in the vehicle group, 99.7% in the no shape, and 99.9–100% in all other patterned groups (Figure 1A). Applying hydrogel with no shape, the WCR slightly increased than the vehicle group, improving from 65.1% to 70.5% at day 7. However, the hydrogels with wave and line patterns demonstrated a more significant increase, reaching 78.2% and 76.8%, respectively. As shown in Figure 1B, the micro-patterned hydrogel patch groups exhibited a higher WCR than all other groups, over 90% at day 7. The fast recovery in the checks micro-patterned group suggests minimal wound fluid drainage, infection, or inflammation during the wound healing phase, further emphasizing its potential as an effective wound dressing.

The measured WCR data over 14 days were nonlinearly fitted to a time-dependent dynamic process using a linear exponential function of the form 1 − exp(−t⁄τ) (Supplementary Figure S1). Table 1 summarizes the results, including the time constant (τ), which represents the number of days required to reach 63.2% of WCR based on the exponential function model (Supplementary Figure S1). Within the same group, larger micro-pattern sizes and wider spacing resulted in a larger time constant, whereas smaller micro-patterned hydrogel exhibited a smaller time constant. Although all micro-patterned hydrogels had the same planar area, differences in wound healing rates were likely due to the increased surface area and volume in the vertical direction. When comparing wound recovery across groups, the time constant was lowest for the checks-patterned hydrogel, followed by the waves and lines patterns. This suggests that the step differences in the structure and increased volume of the micro-patterned hydrogel positively influenced WCR. The results of nonlinear fitting for three groups (vehicle group without hydrogel patch, no shape group with flat hydrogel patch without any pattern, and the checks micro-patterned hydrogel patch group) revealed significant differences in wound healing efficiency. The time constants for the control and no-shape groups were 6.7 days and 5.8 days, respectively. In contrast, the checks micro-patterned hydrogel exhibited a significantly lower time constant of 2.7 days, indicating faster wound healing. The line and wave patterns followed with time constants of 4.0 days and 3.8 days, respectively. Overall, the checks micro-patterned hydrogel demonstrated superior wound healing efficacy compared to other groups.

### 2.2. Hydrogel Improves the Expression of COl1A1 Protein in the Skin

The COL1A1 gene regulates the expression of large molecules called type 1 collagen (Col1A), a key extracellular matrix protein involved in tissue repair and regeneration. In our western blot analysis, we observed upregulated Col1A propeptide expression in groups treated with micro-patterned hydrogel patch. To assess this effect, we analyzed four different types of micro-patterned hydrogel patches (no shape, waves, lines, and checks), at two different time points (day 7 and day 14) using skin samples for Col1A expression.

**Figure 2:**
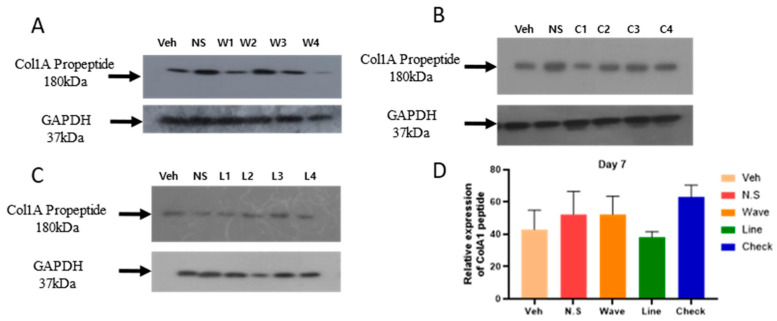
Western blot showing expression of type 1 collagen (Col1A) across different hydrogel patterns at day 7. The expression of Col1A in (**A**) waves hydrogel pattern, (**B**) checks hydrogel pattern, (**C**) lines hydrogel pattern, and (**D**) the total band signals strength of different hydrogels represented in the bar graph. No statistically significant differences in the total band signals strength, Tukey HSD ANOVA. Veh: Vehicle; N.S.: No shape.

At day 7, the expression of Col1A propeptide was upregulated in the waves micro-patterned hydrogel group (Figure 2A) and lines micro-patterned hydrogel group (Figure 2C), whereas it was downregulated in the checks micro-patterned hydrogel group (Figure 2B) compared to the vehicle group. Relative expression analysis revealed that Col1A expression was highest in the checks micro-patterned hydrogel group compared to the waves, lines, no-shape, and vehicle groups, although the difference was not statistically significant (Figure 2D).

**Figure 3:**
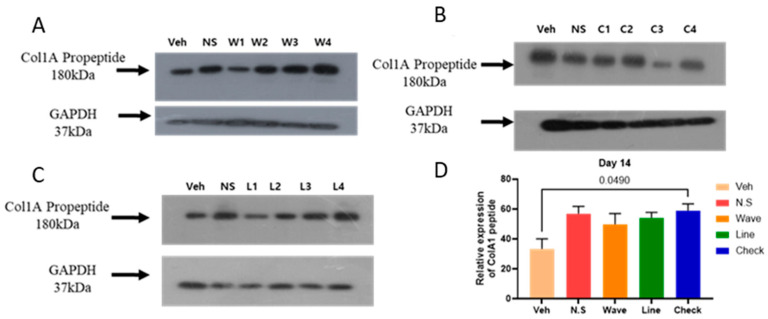
Western blot showing expression of type 1 collagen (Col1A) across different hydrogel patterns on day 14. The expression of Col1A in (**A**) waves hydrogel pattern, (**B**) checks hydrogel pattern, (**C**) lines hydrogel pattern, and (**D**) the total band signals strength of different hydrogels represented in bar graph. A statistically significant difference in the total bands signals strength at *p* > 0.05, Tukey HSD ANOVA. Veh: Vehicle; N.S.: No shape.

After 14 days, the expression of Col1A propeptide was upregulated in all micro-patterned hydrogel groups, including the waves micro-patterned hydrogel (Figure 3A), checks micro-patterned hydrogel (Figure 3B), and lines micro-patterned hydrogel (Figure 3C), compared to the vehicle group. Relative expression analysis indicated that Col1A expression was significantly higher (*p* > 0.05) in the checks micro-patterned hydrogel group compared to the vehicle group (Figure 3D). Additionally, Col1A expression was also upregulated in the waves, lines, and no-shape hydrogel groups, the increase was not statistically significant compared to the vehicle group. Overall, after 14 days, Col1A expression was notably higher in the checks micro-patterned hydrogel group, suggesting enhanced wound healing properties compared to the waves and lines micro-patterned hydrogel groups.

### 2.3. Discussion

The skin serves as an anatomical barrier but is highly susceptible to damage from various external risk factors. The duration required for wound healing varies depending on the extent and depth of the damage, and the management of the injured site during the healing process is considered a critical factor influencing the healing period. To address this, various wound dressings, such as hydrogels, have been developed. Extensive research has focused on hydrogel materials and the drug delivery systems incorporated within hydrogels to promote wound healing. However, comparative studies on the effects of hydrogel micro-patterns remain insufficient. Therefore, this study aimed to fabricate hydrogel patches with various micro-patterns and conduct a comparative analysis of their performance. Our findings demonstrate that hydrogel patches with specific micro-patterns significantly improve wound healing rates, with check patterns showing superior performance. The increased Col1A expression further supports enhanced healing, particularly in the check-patterned hydrogel group, suggesting its potential reliability for clinical applications. These results highlight the importance of hydrogel micro-architecture in wound healing, underscoring the need for further research on long-term effects and the integration of bioactive agents to optimize wound care.

Effective wound dressings should isolate the injured area from the external environment, protecting it from physical, biological, and chemical risk factors, while promoting re-epithelialization. Among various wound dressings, hydrogels are widely recognized for their hydrophilic properties and high water content, which help create a moist environment conducive to healing [20,21]. This study expands our understanding by evaluating distinct micro-patterned hydrogels and their implications for wound healing rates and overall recovery. The moist environment provided by hydrogels prevents wound dehydration, reduces pain, and promotes faster tissue regeneration [22]. Additionally, hydrogels can be engineered to have various properties, such as antimicrobial activity, and anti-inflammatory properties, which further enhance their effectiveness as wound healing [23]. In another hand, infection prolongs the inflammatory stage of wound healing, leading to increased wound fluid drainage. [24,25]. However, in our study, no wound fluid drainage was observed, indicating the absence of infection and inflammation during the healing process.

Research suggests that both the structure (organization of shape and size) and the pattern (arrangement of structures) of hydrogels are critical factors for improving wound healing outcomes. For instance, strategies such as photo-crosslinking hydrogels using visible light [26] and fabricating 3D structured hydrogels [27] have been shown to enhance the healing process by promoting cellular adhesion, proliferation, and migration. These studies emphasize that tailored hydrogel designs can influence biological responses, thereby facilitating optimal wound recovery. The results of this study illustrate that the application of hydrogel patches with micro-patterns significantly accelerated the wound healing process compared to controls and patches without micro-patterns. Among the micro-patterns tested, the checks pattern emerged as the most effective, demonstrating a healing rate approximately 2.9 times faster than the control group and 2.5 times faster than the non-patterned hydrogel patch. This significant improvement underscores the potential of checking micro-patterned hydrogels in promoting rapid wound closure. The likely mechanism behind this enhancement could be the increased surface area and volume in the vertical direction, which may facilitate better moisture retention and higher cellular interactions, which may facilitate better moisture retention and higher cellular interactions, thereby promoting faster tissue regeneration [22].

In wound healing, fibroblasts differentiate into myofibroblasts to contract the wound area and contribute to tissue regeneration by synthesizing collagen and producing ECM proteins such as proteoglycans, elastin, and fibronectin [28,29]. Fibroblasts, which play a crucial role in wound healing, migrate to the wound site during the early stages of skin incision through chemotaxis, activated by platelet-derived growth factor, transforming growth factor-beta, and other signaling factors [30]. Previous studies have reported that fibroblasts migrating and proliferating at the skin incision site promote wound healing through interactions with M2 macrophages, influenced by the physical properties of hydrogels such as stiffness, flexibility, and surface patterns [31,32]. In particular, previous studies demonstrating that patterned substrates influence fibroblast alignment suggest that wound healing can be regulated by hydrogel micro-patterns [33,34]. This suggests that the surface patterns in contact with the wound site can significantly influence the wound-healing process. In addition, the wound healing rate also depends on the shape of the wounds (circular, triangle, rectangle, and square) [35]. In this study, we hypothesize that faster wound healing is associated with better cell migration along the wound edge. Based on this hypothesis, we have chosen a circular wound incision, which showed better results of WCR.

When comparing different micro-patterns (lines, wave, and checks), the checks pattern consistently showed superior performance across all measured intervals. This suggests that the geometry and dimensionality of the pattern play crucial roles in modifying the wound microenvironment to promote healing [36]. The checks micro-patterned stepped structure may provide additional mechanical support and a larger surface for cell adhesion and proliferation [37]. Furthermore, within the same pattern type, the healing rate improved with decreasing pattern interval width, highlighting the importance of micro-pattern density in optimizing wound healing outcomes. The overall trend observed within and across groups indicates that smaller, more closely spaced micro-patterned were more effective. Previous studies have explored different shapes (circular, triangle, rectangle, and square) [35], different arrangements like photo-crosslinking by visible light [26], and 3D structural hydrogel for wound healing [27].

Another key aspect of wound healing is moisture retention in the hydrogel. Maintaining a moist environment is critical for effective wound healing, as it promotes cellular activities such as migration, proliferation, and extracellular matrix deposition. Studies have shown that moisture-retentive dressings, such as hydrogels, accelerate WCR by preventing desiccation and supporting optimal hydration levels, which are essential for epithelialization and granulation tissue formation [38]. Conversely, wounds exposed to dry conditions often experience delayed healing due to impaired cellular functions and reduced tissue regeneration.

Type I collagen plays a crucial role in each stage of wound healing by providing structural support, regulating cellular signaling, and promoting tissue regeneration. It is recognized as an essential factor in assessing the quality and rate of wound healing [39]. Therefore, the proper synthesis and secretion of Type I collagen are critical for the successful progression of the wound repair process [40,41]. The biological mechanisms regulating Type I collagen are pivotal in wound management and therapeutic development. In this context, Type I collagen has been widely utilized as a functional material in hydrogels [42,43,44]. Type I collagen, coded by the COL1A1 gene, is critical in wound repair. Its expression serves as a marker for cellular proliferation and tissue regeneration during the wound healing process. In this study, the Western blot analysis of COL1A1 expression corroborated the WCR findings, and elevated COL1A1 levels were evident in most of the micro-patterned hydrogel patch groups compared to the controls, especially noticeable by day 14. Specifically, increased collagen expression in the waves and checks patterns aligned with their enhanced wound healing rates, further validating the efficacy of these micro-patterned hydrogels. Interestingly, while the wave pattern indicated higher collagen expression, the checks pattern showed more consistent performance across multiple analyses, making it potentially more reliable for clinical applications.

The clinical applicability of these findings suggests that incorporating specific micro-patterned hydrogels into wound care protocols could significantly improve patient outcomes by accelerating healing times and reducing the frequency of dressing changes. This would not only lower overall healthcare costs but also improve the quality of life for patients [45]. The biodegradability and multifunctional capabilities of materials such as PVA, PEG, and chitosan used in creating these micro-patterned hydrogels, further enhance their practical utility across various medical fields, including tissue engineering and drug delivery. In addition, to enhance these findings, future studies should explore the long-term effects of micro-patterned hydrogel dressings on different types of wounds, such as chronic wounds or deeper tissue injuries. Additionally, investigating the integration of bioactive agents within these micro-patterned hydrogel structures could provide insights into combined therapeutic strategies for even more efficient wound management. Finally, understanding the molecular mechanisms by which these microstructures influence cell behavior and tissue regeneration will be pivotal for further optimizing these materials for clinical use. This study provides compelling evidence that micro-patterned hydrogel dressings, particularly those with smaller and densely packed patterns like the check arrangement, significantly enhance the wound healing process. These results pave the way for the development of more effective and application-specific wound dressing materials in the future.

## 3. Conclusions

In conclusion, the micro-patterned hydrogels demonstrated a pattern-dependent effect on wound healing efficacy. Specifically, the checks micro-patterned hydrogel patches significantly enhanced the wound healing rate, suggesting that a more structured and closely packed design supports more effective healing. Additionally, there was a notable increase in Col1A protein expression after 14 days, particularly in the checks pattern, indicating that specific designs not only promote faster closure of wounds but also improve the underlying structural tissue regeneration. We also found that the depth of the grooves may impact the WCR, and further research should be conducted to comprehensively evaluate its role in wound healing. Overall, these findings suggest that hydrogel shape and pattern play critical roles in enhancing wound healing and tissue repair processes. Further studies could optimize these parameters for targeted clinical applications in skin wound management and explore the long-term effects and integration of bioactive agents within these micro-patterned hydrogels.

## 4. Materials and Methods

### 4.1. Nickel (Ni) Stamp

#### 4.1.1. Structure

The micro-patterned hydrogel surface of the wound-healing patch coating material was generated using a hot-embossing process with a Nickel (Ni) stamp. To evaluate the effectiveness of wound healing properties of the micro-patterned hydrogel, three types of microstructure patterns were selected: waves, lines, and checks, with the widths ranging from 100 to 500 µm, while the height of the micro-patterned hydrogel was fixed at 50 µm. The overall size of the wound healing mold was 25 mm × 25 mm (Table 2).

#### 4.1.2. Manufacturing Process

**Figure 4:**
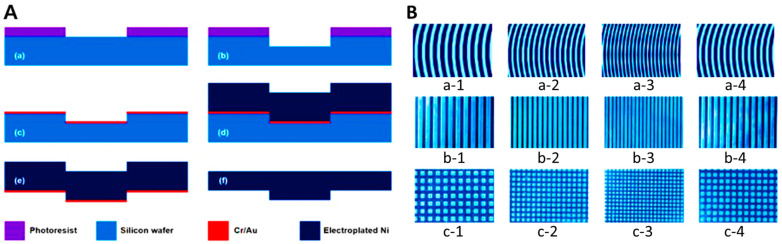
Nickel stamp manufacturing process using an electroplating procedure. (**A**(**a**)) Planarization using PR patterning, (**A**(**b**)) silicon dry etch, (**A**(**c**)) PR removal and Cr/Au deposition, (**A**(**d**)) Ni electroplating, (**A**(**e**)) lower silicon wet etch, (**A**(**f**)) CMP process. A photograph of the Ni stamp of three different patterns, (**B**(**a-1**–**a-4**)) waves, (**B**(**b-1**–**b-4**)) lines, and (**B**(**c-1**–**c-4**)) checks (width spacing of patterns 1: 500 µm, 2: 200 µm, 3: 100 µm, 4: 250 µm).

The Ni stamp was fabricated using an electroplating process (Figure 4). The process began with the application of a photoresist (PR, AZ GXR 601, Merck Performance Materials, Darmstadt, Hesse, Germany) on a silicon wafer, followed by spin-coating at 4000 rpm for 35 s. After curing at 90 °C for 1 min, UV light with a wavelength of 365 nm was irradiated for 2 s, and the PR was developed with 2.38 wt% of tetramethylammonium hydroxide (TMAH). A hard-curing process at 100 °C for 1 min completed the PR patterning (Figure 4A(a)). A front of the silicon wafer was etched by 50 µm using the deep reactive ion etching process, and residual PR was removed (Figure 4A(b)). Following the silicon processing, Cr (chromium) and Au (gold) seed layers for Ni plating were deposited using an e-beam evaporator to thicknesses of 300 Å and 1000 Å, respectively (Figure 4A(c)). A Ni structure with a thickness of 50 µm was then fabricated through a Ni electroplating process over 14 hr, using a solution containing 600 g/L of Ni(SO)_4_·6H_2_O (Nickel(II) sulfate hexahydrate), 10 g/L of NiCl_2_·6H_2_O (Nickel(II) chloride hexahydrate), and 40 g/L H_3_BO_3_ (Boric acid). The current density and temperature were maintained at 7.5−8.5 mA/cm^2^ and 45 °C, respectively (Figure 4A(d)). After the electroplating process, the residual silicon wafer was removed by a wet etching process with 20 wt% TMAH (Figure 4A(e)). Finally, the Cr and Au seed layers were removed, and the fabrication process of the Ni stamp was completed using a chemical mechanical polishing (CMP) process for polishing and smoothing the upper Ni structure (Figure 4A(f)). A photograph of the Ni stamp of three patterns, waves (Figure 4B(a-1–a-4)), lines (Figure 4B(b-1–b-4)), and checks (Figure 4B(c-1–c-4)), was produced. All chemicals were purchased from Sigma Aldrich (Saint Louis, Missouri, USA) unless otherwise stated.

### 4.2. Wrinkle Shape Replication Process Using a Hot-Embossing Process

#### 4.2.1. Polymer Preparation

In this study, a silicone elastomer (DOW CORNING 7-9700 Soft Skin Adhesive, Midland, MI, USA) was used to manufacture the micro-patterned hydrogel wound dressing. This elastomer was selected for its biocompatibility, established adhesive properties, and proven compatibility in biomedical applications, as documented by the manufacturers and supported by their expertise in wound dressings. Part A and Part B of the silicone elastomer were mixed in a 1:1 ratio using a mixing nozzle (1/2″ × 24 elements, IMS Company, Chagrin Falls, OH, USA).

#### 4.2.2. Manufacturing Process

**Figure 5:**
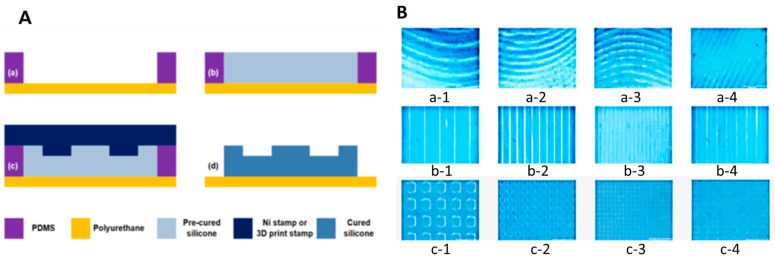
The process of manufacturing a wound dressing with fine wrinkles. (**A**(**a**)) PDMS mold fabrication, (**A**(**b**)) silicone application and weak curing, (**A**(**c**)) microstructure replication using hot-embossing process, (**A**(**d**)) Ni stamp and PDMS mold removal. A photograph of the microstructure was replicated on top of the wound dressing, (**B**(**a-1**–**a-4**)) waves, (**B**(**b-1**–**b-4**)) lines, and (**B**(**c-1**–**c-4**)) checks (width spacing of patterns 1: 500 µm, 2: 200 µm, 3: 100 µm, 4: 250 µm).

The process of manufacturing the micro-patterned hydrogel wound dressing with fine wrinkles. First, a frame made of polydimethylsiloxane (PDMS) material that can fix wound dressings with a certain thickness and external appearance was manufactured. In a Petri dish (150 × 20 mm, SPL, Pocheon-si, Gyeonggi-do, Republic of Korea), PDMS polymer and curing agent are mixed in a ratio of 10:1; the air bubbles were removed at room temperature and then cured at 90 °C for 30 min using a hotplate (Hi-1000, AS-ONE, Osaka, Japan). After separating the cured PDMS from the Petri dish, cut it into a size of 25 mm × 25 mm, and put on the polyurethane film to complete the PDMS frame for wound dressing (Figure 5A(a)). After applying 1.1 mL of silicone elastomer prepared in advance to the PDMS frame, perform a curing process at 90 °C for 30 min using a hotplate (Figure 5A(b)). Place the Ni stamp on the cured silicone elastomer and thermo-compress at 430 kPa pressure and 150 °C for 20 min using a heating press (COAD-1004, Ocean Science, Pocheon-si, Gyeonggi-do, Republic of Korea) (Figure 5A(c)). After separating the Ni stamp and removing the PDMS mold, the micros-patterned replication process was completed (Figure 5A(d)).

#### 4.2.3. Production Result Analysis

An image taken using an optical microscope (KH-7700, HIROX, Suginami-ku, Tokyo, Japan) of the micro-patterned replicated on top of the wound dressing patches of three different patterns waves (Figure 5B(a-1–a-4)), lines (Figure 5B(b-1–b-4)), and checks (Figure 5B(c-1–c-4)).

### 4.3. Animal Study

#### 4.3.1. Experimental Animals

Seven Sprague-Dawley rats (SD male, 7-week-old, 230 ± 10 g) were used in this study under the IACUC guideline of Inje University, Korea. Briefly, rats were acclimatized for a week in a controlled environment with a photoperiod of 12/12 h dark/light cycle, at a temperature of 22 ± 0.5 °C and humidity of 55 ± 5%. During the acclimatization period, standard laboratory chow and autoclaved tap water were provided ad libitum.

#### 4.3.2. Production of Secondary Union Skin Damage (Wound) Animal Model

**Figure 6:**
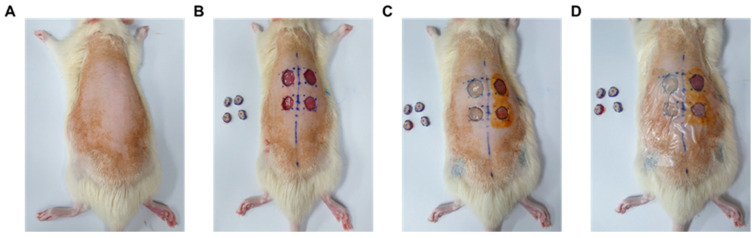
Animal model of secondary fusion skin incision and hydrogel application pattern. Preparation of sterilized skin by removing hair (**A**), preparation of wounds on sterilized skin by incisions (**B**), application of microstructure hydrogels patched (**C**), and closing of wounds with Tegaderm^TM^ (**D**).

After 1 week of acclimatization, (8-week-old, 240 ± 10 g) experimental animals were anesthetized with a mixture of 3% isoflurane (Hana Pharmaceutical, Hwaseong-si, Gyeonggi-do, Republic of Korea) and atmospheric air through a flowmeter system-based inhalation anesthetic (Harvard Apparatus, Holliston, MA, USA). For wounds by skin incision, first, dorsolateral position hair was removed using a sterilized scalpel (Figure 6A). A total of 4 wounds of 1 cm diameter incision on sterilized skin were made with scissors, 2 left and 2 right (Figure 6B). Thereafter, the wound was disinfected with a 10% povidone-iodine solution. Animals with skin incisions were randomly classified into a control group and micro-patterned hydrogel patch application groups. The prepared micro-patterned hydrogel patch was applied to the wound immediately after skin incision surgery (Figure 6C) and fixed with Tegaderm^TM^ (3M, Saint Paul, MN, USA) (Figure 6D). The experiment was terminated at the time point of 100% wound healing rate (14th day). During wound healing time, animals were kept in the same controlled environment in which temperature (22 ± 0.5 °C), humidity (55 ± 5%), and 12 h of day and night atmosphere, and food and water were provided ad libitum. The experimental procedure was conducted with the approval of the Inje University Animal Experimentation Ethics Committee (IACUC) (approval number: Inje 2021-005). All experimental animals were raised in the same conditions.

#### 4.3.3. Experimental Group and Micro-Patterned Hydrogel Patch Application

Animals were divided into a total of fourteen experimental groups: one vehicle group (only skin incision) and thirteen treatment groups (skin incision treatment with micro-patterned hydrogel). Thirteen different types of hydrogel patches, each with different patterns and intervals between patterns, were fixed with Tegaderm (3M) and applied to the wounds (Table 2). The shapes explored include no shape, waves, lines, and checks, each with varying intervals ranging from 100 µm to 500 µm. The patterns (waves, lines, and checks) and the combined intervals (500 µm + 200 µm + 100 µm + 250 µm) were analyzed to compare the effects of these geometrical configurations on wound healing. The pattern-structure approach allows for a comprehensive examination of how different micro-pattern configurations and spacing influence the efficacy of micro-patterned hydrogel patches in promoting wound recovery. Concerning the recovery, patches were replaced every 3rd day during the study period. The patches were replaced every third day during the study period. The skin samples were collected at two time points: day 7 (mid-study) and day 14 (study termination) for further analysis.

#### 4.3.4. Wound Closure Rate

Pictures of the wounds were captured at three different time points: immediately after skin incision surgery (day 1) and during tissue collection on day 7 and day 14. These images were analyzed using ImageJ software (ver. 1.8; NIH, Bethesda, MD, USA) to calculate the WCR. WCR, expressed as a percentage, was determined using the following formula (Equation (1)).

(1) $$WCR\left(\%\right)=\left(\frac{A_{p}-A_{e}}{A_{p}}\right)\times 100$$

where *A_p_* represents the wound area on day 1 (immediately after surgery), and *A_e_* represents the wound area at the time of tissue collection (day 7 or day 14).

#### 4.3.5. Western Blot

The skin samples collected on day 7 and day 14 were processed for protein expression by western blot. A total of 4 wound incisions (2 + 2 sample sets) were created on rat skin on day 1. Notably, 7 days later, the WCR was calculated and 2 samples were collected for the western blot analysis. The remaining 2 samples were collected on day 14 for additional western blot sampling after the WCR was calculated. Total protein was extracted in a fixed weight/volume per sample using RIPA buffer (Sigma-Aldrich, St. Louis, MO, USA), containing protease inhibitors (Roche Diagnostics, Rotkreuz, Canton of Zug, Switzerland), and homogenized with an ultrasonic liquid processor (Sonics & Materials, Inc., Newtown, CT, USA). The tissue homogenates were centrifuged at 14,000 rpm for 30 min at 4 °C to remove the insoluble materials, and the resultant supernatants were stored at −80 °C until further use. The protein content of the supernatants was determined using a protein assay kit (Bio-Rad, Hercules, CA, USA). The samples were subjected to denaturation at 100 °C in Laemmli sample buffer (Bio-Rad, Hercules, California, USA) containing 5% β-mercaptoethanol. 100 µg of protein was resolved in an SDS-PAGE gel and transferred to a PVDF membrane (Millipore, Billerica, MA, USA) using a Mini Trans-Blot^®^ Electrophoretic Transfer Cell device (Bio-Rad, Hercules, CA, USA). Membranes were blocked with 1× Tris-buffered saline (1× TBS) containing 0.1% Tween 20 (TBST) and 5% BSA (Sigma-Aldrich, St. Louis, MO, USA) or non-fat dried milk (Nestlé, Vevey, Vaud, Switzerland). They were then incubated overnight with anti-Col1A polypeptide (Abcam, Cambridge, UK) and anti-beta actin antibodies (Cell Signaling Technology, Danvers, MA, USA) (1:1000 dilution in 1× TBST containing 3% BSA or non-fat skim milk) at 4 °C. After overnight incubation, membranes were washed with 1× TBST three times. Then, membranes were incubated for 90 min with the appropriate horseradish peroxidase-conjugated anti-IgG secondary antibodies (Santa Cruz Biotechnology, Santa Cruz, CA, USA) (1:2000 dilution in TBST containing 1% non-fat skim milk or BSA). Finally, the immunoreactive bands were detected using an imaging system (Bio-Rad, Hercules, CA, USA) and ECL reagent (Thermo Fisher Scientific, Waltham, MA, USA). The band intensity was measured with inbuilt software (v.1.42q).

#### 4.3.6. Statistical Analysis

All analyses were performed using SPSS (statistical package software, ver. 27.0, IBM, New York, NY, USA). The results derived between experiments were represented as mean ± standard deviation and statistical significance was considered statistically significant when the *p*-value was less than 0.05 by conducting an independent sample *t*-test.

## Figures and Tables

**Table 1:** The time constant of the wound healing.

Hydrogel Microstructure	Average of Time Constant (Day)
Veh ^1^ (Control)	6.7
N.S. ^2^ (Flat)	5.8
Waves	4.0
Lines	3.8
Checks	2.7

^1^ Vehicle; ^2^ No shape.

**Table 2:** Pattern of microstructure hydrogel patch coating material.

Pattern	Flat	Wave	Wave	Wave	Wave	Line	Line	Line	Line	Check	Check	Check	Check
Code	N.S.	a-1	a-2	a-3	a-4	b-1	b-2	b-3	b-4	c-1	c-2	c-3	c-4
Interval	-	500 μm	200 μm	100 μm	250 μm	500 μm	200 μm	100 μm	250 μm	500 μm	200 μm	100 μm	250 μm

## Data Availability

The raw data supporting the conclusions of this article will be made available by the authors upon request.

## Supplementary Materials

The following supporting information can be downloaded at: https://www.mdpi.com/article/10.3390/gels11040239/s1, Figure S1: Wound closure rate nonlinearly fitted for the time-dependent dynamic process using a linear exponential function 1 − exp(−t⁄τ). Where τ is a characteristic time constant. Veh: Vehicle; N.S.: No shape, and C: Checks micro-patterned.
