# Supplementary material for: Comparison of Wound Healing Effects of Different Micro-Patterned Hydrogels on the Skin of Secondary Intention Rat Model
Source: Gels. 2025 Mar 24;11(4):239. doi: 10.3390/gels11040239 (PMC12026865; doi:10.3390/gels11040239)

Wound healing: western blot; Col1A Propeptide (Lines)

Lines

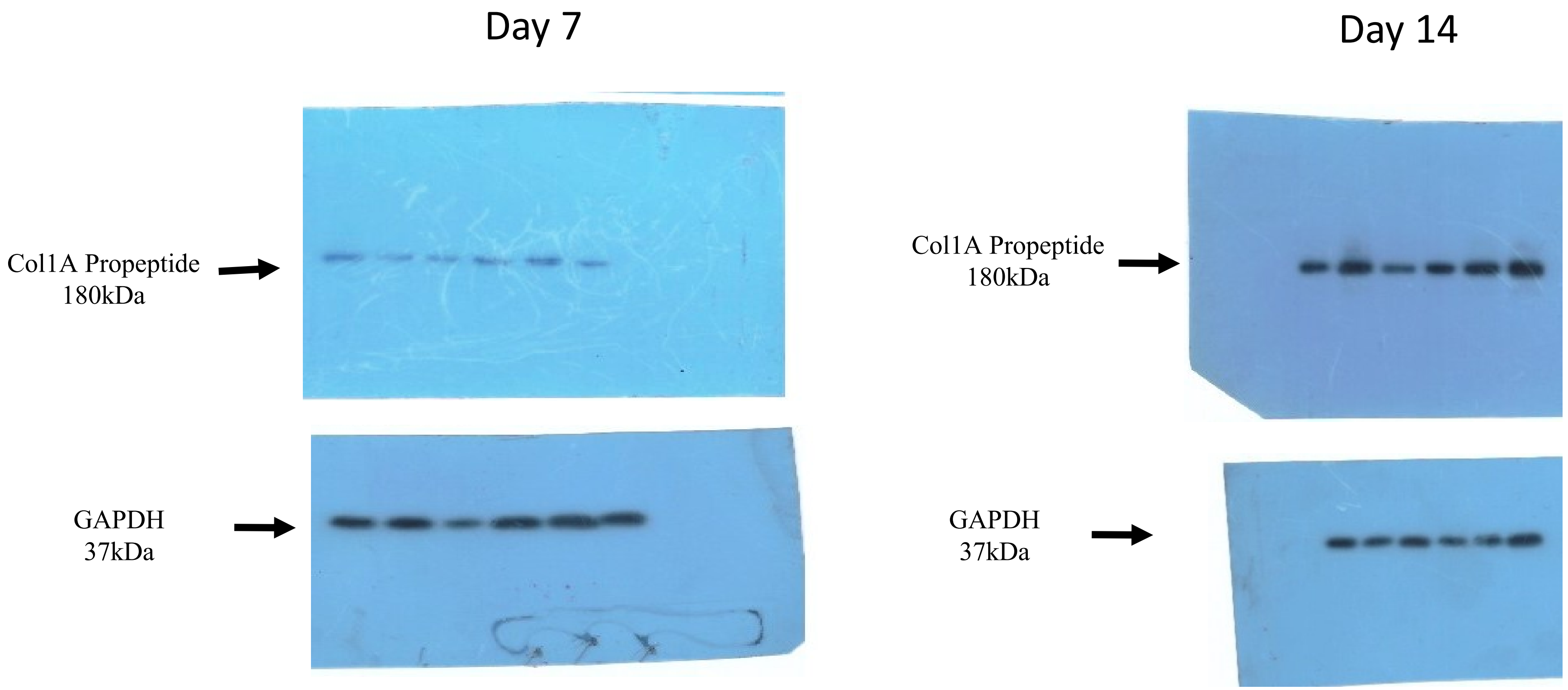

Wound healing: western blot; Col1A Propeptide (Checks)

Checks

Day 7

Col1A Propeptide
180kDa

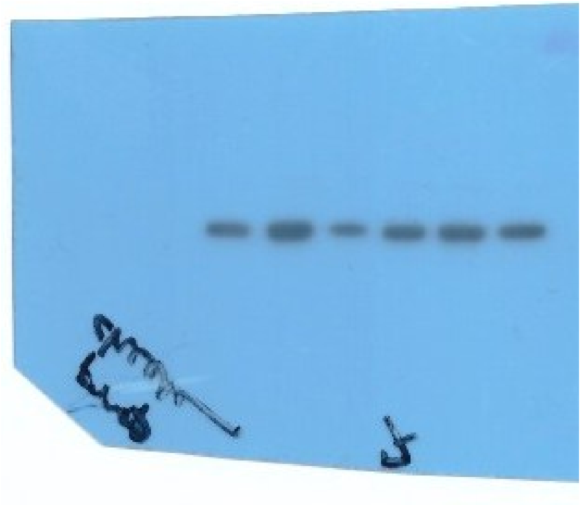

GAPDH
37kDa

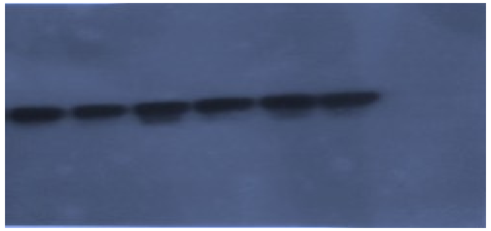

Day 14

Col1A Propeptide
180kDa

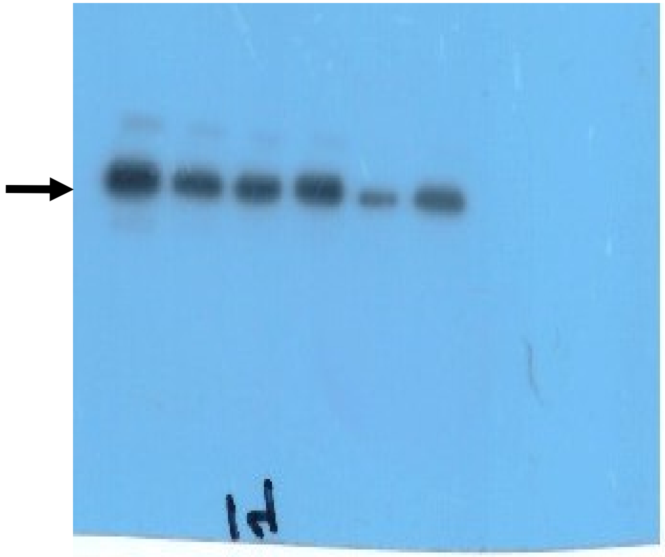

GAPDH
37kDa

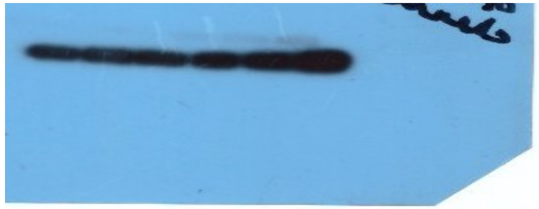

Wound healing: western blots; Col1A Propeptide (Waves)

Waves

Day 7

Col1A Propeptide
180kDa

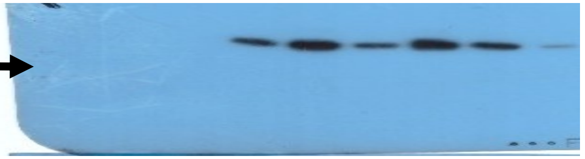

GAPDH
37kDa

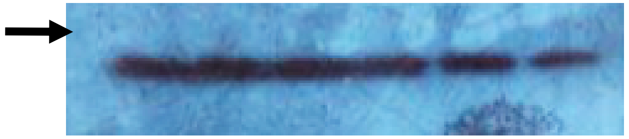

Day 14

Col1A Propeptide
180kDa

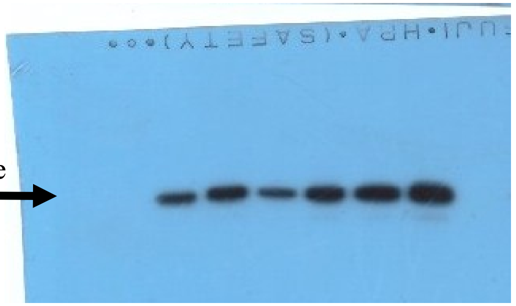

GAPDH
37kDa

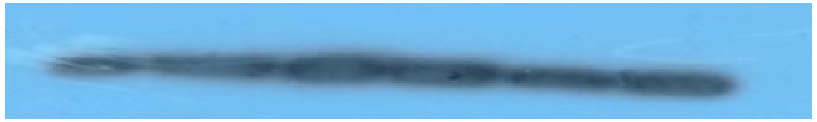

Supplement: Supplementary file 1 [file gels-11-00239-s001.zip › gels-3539300-supplementary.pdf]
